# Exploring emotional intelligence through emotional journaling: a qualitative study of young students

**DOI:** 10.1080/17482631.2026.2688506

**Published:** 2026-08-13

**Authors:** Sleiman El Hajj, Myriam El Khoury-Malhame, Rita Doumit, Roni Chaaya, Maria-Jose Sanchez-Ruiz

**Affiliations:** a Department of English and Creative Arts, School of Arts and Sciences, Lebanese American University, Beirut, Lebanon; b Department of Psychology and Education, School of Arts and Sciences, Lebanese American University, Beirut, Lebanon; c Alice Ramez Chaghoury School of Nursing, Lebanese American University, Beirut, Lebanon; d Department of Personality Psychology, Assessment, and Psychological Treatment, School of Psychology, Universidad Nacional de Educación a Distancia (UNED), Madrid, Spain

**Keywords:** Emotion diary study, trait emotional intelligence, emotional competences, adolescents, mental health, vulnerable youth

## Abstract

**Background:**

This paper examines the emotional experiences of vulnerable Lebanese youth in light of the context of prolonged and uninterrupted social, political, and economic crises impacting Lebanon in recent years.

**Method:**

Conducted as part of the “Yes to Emotions in Youth” (YEY) program, which focused on emotional intelligence (EI) training at a semi-private school in Lebanon, the research analyzes the emotional diaries from 145 students (98 girls) aged 15–17. These diaries were analyzed using thematic analysis informed by Colaizzi’s phenomenological method. The study adopts the framework of trait Emotional Intelligence and utilized Brasseur et al. (2013) model of five emotional competences: identification, understanding, expression, regulation, and utilization of emotions.

**Results:**

Four overarching themes were discerned: (One) Emotional identification: unpleasant emotions and triggers; (Two) Emotional identification: pleasant emotions and triggers; (Three) Coping and emotional regulation: maladaptive practices; and (Four) Coping and emotional regulation: adaptive practices. While the sampled population is, in general, attuned with, and shows competence in identifying emotions and their triggers, many of the youth participants struggled with regulating these emotions adaptively.

**Discussion:**

The findings highlight the disparate layers of EI-informed coping in relation to emotion identification and regulation among youth grappling with the precarity of daily life.

## Introduction

Emotional competence plays a pivotal role in diverse life domains, with particular emphasis on emotion regulation, which has been consistently linked to enhanced psychological well-being (Ford & Gross, 2018). These competences become especially critical in contexts characterized by chronic psychological stress, such as environments afflicted by ongoing conflict (Santiago, 2024; Šimunjak, 2023). Lebanon, for instance, has endured a succession of compounding crises since 2019, including economic collapse, political gridlock, social unrest, the devastating 2020 Beirut port explosion, and the global COVID-19 pandemic, while still grappling with the long-term aftermath of the 1975–1990 civil war (El Hajj, 2021a, 2023). Within such destabilizing conditions, emotional competence may be compromised, especially during adolescence. This is a developmental period marked by rapid and profound physical, emotional, social, and cognitive transformations that scaffold the transition to adulthood. Adolescents actively engage in identity exploration, cultivate increasingly complex relationships, and strive for autonomy and self-definition (Blakemore, 2018). At the same time, they become more susceptible to emotional dysregulation and psychosocial stressors, particularly in unstable environments such as Lebanon (Brown et al., 2020; Jordans et al., 2023). Hence, the ability to understand, manage, and utilize emotions, core elements of emotional competence, is especially consequential during adolescence, as it underpins youth psychological adjustment and well-being.

Within this developmental context, Emotional Intelligence (EI) offers a valuable framework for understanding how adolescents perceive and manage their emotional world. Although the term was popularized by Goleman (1995), the scientific conceptualization of EI originated with Salovey and Mayer (1990), who defined the construct as a set of emotion-related cognitive abilities, including perceiving, understanding, and managing emotions, typically assessed through maximum-performance tasks. In addition, Bar-On (1997) proposed a broader framework incorporating emotional and social competences within a mixed model. The present study adopts the trait Emotional Intelligence (trait EI) framework. Better assessed through self-report measures, this framework reflects individuals’ emotion-related self-perceptions and behavioral dispositions embedded within personality hierarchies (Petrides et al., 2007). In simple terms, trait EI concerns people’s perceptions of their emotional world and their perceived ability to understand, regulate, and express emotions effectively.

Trait EI has been linked to psychological adjustment, well-being, and adaptive functioning, and has shown particular relevance in educational contexts, where it predicts better school adjustment, prosocial behavior, academic engagement, and performance-related outcomes (Mavroveli & Sanchez-Ruiz, 2011; Petrides et al., 2016). In addition, higher trait EI has been associated with fewer unauthorized absences, lower likelihood of rule violations, higher creativity, and improved academic performance (Petrides et al., 2018). All these outcomes highlight its importance as a key non-cognitive factor supporting students’ academic and emotional development. Recent systematic review evidence supports the link between trait EI and well-being among adolescents (Mancini et al., 2024). Indeed, a growing body of empirical research supports the association of trait EI with a wide range of adaptive outcomes, including not only enhanced academic and occupational performance, but also better interpersonal functioning, and improved mental and physical health (Petrides et al., 2016).

Similarly, in Lebanon, higher trait EI has been associated with improved academic performance, engagement, and well-being (Sanchez-Ruiz & El Khoury, 2019; Sanchez-Ruiz et al., 2024). Other studies in the Lebanese context have demonstrated that higher trait EI correlates with indicators of psychological well-being and serves as a protective factor against various forms of psychopathology (Sanchez-Ruiz & El Khoury, 2019; Sanchez-Ruiz et al., 2021; Zeeni et al., 2018), which is especially important in this developmental stage. Adolescents with high trait EI are more likely to employ adaptive coping strategies—such as problem-solving, positive reframing, and seeking social support—that mitigate stress and foster resilience (Mikolajczak & Luminet, 2008). In contrast, low levels of trait EI have been associated with maladaptive coping mechanisms, including avoidance and emotion-focused strategies, which can exacerbate psychological distress (Zeidner et al., 2004). These patterns have been further supported in Lebanese youth, where trait EI has shown a positive association with meaning-centered coping and a negative relationship with maladaptive responses to stress (Sanchez-Ruiz et al., 2021).

### EI, and social and emotional learning (SEL), in Lebanon

Despite increasing global interest in youth emotional and social intelligence (Jose & Thomas, 2024), youth EI-based research in the Middle East remains limited (Nassar et al., 2023), particularly in Lebanon despite its mostly young population. Existing studies tend to be quantitative and psychometric (Badri et al., 2021; El Khoury-Malhame et al., 2026, 2024a; Jordans et al., 2023), with few qualitative explorations of adolescents’ emotional experiences. Indeed, Hejase et al. (2012) offer the only qualitative EI study in Lebanon to date, focusing on business managers rather than youth. To address this gap, the present study, embedded within an EI training program at a Lebanese semi-private school, qualitatively explores the EI competences of adolescent participants, particularly concerning the identification, expression, and regulation of emotions. The findings aim to inform psychosocial interventions and EI training initiatives tailored to Lebanese youth navigating adversity, hence the choice of the Social and Emotional Learning (SEL) baseline informing the study design.

SEL constitutes a comprehensive educational framework aimed at promoting students’ social, emotional, and behavioral competences within school contexts (Weissberg et al., 2015). Globally, SEL-based learning programs are used to provide scaffolded emotional support to vulnerable youth, such as out-of-school children in conflict zones (Turner et al., 2022). Improvements in resilience, self-esteem, and psychological well-being have been reported as key SEL program outcomes addressing youth populations (Brill et al., 2025). Indeed, SEL programs are grounded in developmental and prevention science; they typically target constructs related to self-awareness, self-regulation and interpersonal competences as articulated by the Collaborative for Academic, Social, and Emotional Learning (CASEL). Robust meta-analytic evidence demonstrates that SEL interventions produce significant improvements in social-emotional skills, academic achievement, and psychological well-being, while reducing emotional distress and behavioral problems (Taylor et al., 2017), within their respective educational landscape.

In particular, Lebanon’s education system has faced considerable challenges in recent years due to economic instability, school closures, and political unrest (UNESCO, 2023). These systemic challenges significantly impact youth mental health, leading to increased stress, emotional dysregulation, and reliance on maladaptive coping mechanisms (El Khoury-Malhame et al., 2024b; Fawaz et al., 2023). Given the role of trait EI in supporting psychological well-being, integrating SEL-informed EI training programs into Lebanese schools could provide sustainable, long-term benefits for youth development and can provide an evidence-based foundation for expanding EI training programs in Lebanon, with implications for education policy, youth mental health interventions, and social-emotional development initiatives.

### SEL-based “yes to emotions in youth” (YEY) program

Knowing that emotional self-perceptions can be developed through structured training programs targeting emotional awareness, regulation, and expression, research supports the value of incorporating trait EI into educational interventions, effectively improving emotional functioning, academic adjustment, and psychological well-being. Trait-EI-based interventions are therefore relevant for school-based mental health promotion (Petrides et al., 2016). The present study is embedded within one such youth-led intervention, the “Yes to Emotions in Youth” (YEY) program implemented in Lebanese public schools to promote adolescent mental health among vulnerable 15- to 17-year-olds.

Some psychosocial initiatives, including school-based SEL programs and community mental health initiatives, such as Early Adolescent Skills for Emotions (EASE), have emerged in Lebanon of late (Jordans et al., 2023), but there remains a need for structured, research-based EI training programs tailored to the needs of Lebanese youth. Developed by the World Health Organization, EASE was culturally adapted and implemented in other contexts enduring displacement and adversity, such as Tanzania, Jordan, and Pakistan. While EASE aimed to address symptoms of depression and anxiety among 10- to 14-year-old Syrian and Palestinian refugees as well as Lebanese children exhibiting a high level of psychological distress (Brown et al., 2020), YEY is, to our knowledge, the only intervention to date targeting older Lebanese youths (high schoolers) in an educational setting, rather than younger, even more vulnerable, children. The YEY program in Lebanon leans on empirically supported principles for effective trait-EI training and implementation (Mikolajczak et al., 2009) and has shown preliminary evidence of success in reducing psychological distress and fostering well-being (Sanchez-Ruiz et al., 2024, 2025).

Structurally, YEY is organized according to the Profile of Emotional Competence (PEC) model (Brasseur et al., 2013; Mikolajczak et al., 2015), which encompasses five core competences: identification, comprehension, expression, regulation, and utilization of emotions. Within this framework, trait EI serves as the overarching dispositional lens, while the PEC model provides the structured competence-based map through which EI was fostered during the training and reflected in the emotional diaries. YEY’s approach is rooted in youth-centered, participatory methods, where adolescents actively engage in guided discussions and reflective exercises, such as journaling, to explore and articulate their emotional experiences. The training modules incorporated innovative methods in mindfulness, journaling, visualization, and debate. These practices, in helping participants improve their emotional regulation skills, optimize well-being, quality of life, social interactions, and employability (Nelis et al., 2011). By creating a supportive and non-judgmental environment, the YEY program encouraged young people to openly discuss emotions, understand the impact of their emotional responses, and learn effective strategies for emotional regulation, skills that Zeidner et al. (2012) confirm are crucial for enhancing mental health, academic adjustment, and social integration among vulnerable youth populations.

### Emotional intelligence in YEY’s emotional diaries

Given the study design’s thematic analysis of emotional competences among youth in a conflicted setting, this qualitative approach adds to other diary studies in Lebanon, which have not examined these themes (El Hajj, 2018; Khalaf, 2014), and also to other EI studies which deploy psychometric variables, intraclass coefficients, and/or other quantitative instruments to examine the use of emotion management strategies in daily life within Lebanon (El Khoury-Malhame et al., 2024a, 2026) and beyond (Brans et al., 2013; English et al., 2017; Pekaar et al., 2018). The choice of a qualitative framework, premised on the emotional diary as its main mode of inquiry, is further validated by ongoing interventional EI research in Lebanon (Sanchez-Ruiz et al., 2026), which finds that diary writing in EI training generates, across various quantitative measures for EI acquisition and mental health, more favorable results than interventions that lack a diary component. In apprising and appraising EI competences in practice, the study reveals the boons and shortcomings pertinent to an understudied population’s exploration and awareness of EI and coping strategies.

By adopting a qualitative thematic approach, the study thereby provides process-oriented insight into the lived manifestation of EI competences, complementing the predominantly quantitative literature in the field. Scarce as emotional diaries are in extant scholarship, these are recognized as valuable tools within SEL interventions, aimed at enhancing self-awareness, emotion regulation, and coping mechanisms (Moroń & Biolik-Moroń, 2021; Ohly et al., 2010). Such diaries facilitate the tracking of emotional triggers, the processing of emotional responses, and reflection on coping strategies (Nelis et al., 2009). Knowing that structured journaling has been shown to improve emotional clarity, resilience, decision-making, and psychological well-being (Brans et al., 2013; Pekaar et al., 2018), emotional diaries in the present study were integrated into the YEY training sessions, guiding participants to document triggering events and emotional responses. Participants reflected on how they identified emotions, their perceived causes, expressions in different situations, and methods of regulation. Hence, in providing a structured space to express, conceptualize, and apply the competences introduced during the sessions, the emotional diaries resulting from the intervention served as a reflective tool for self-awareness and a qualitative method for analyzing youth emotional engagement.

While past research confirms that diary writing fosters EI competences, particularly among adolescents who may struggle with self-reflection and emotional processing (Moroń & Biolik-Moroń, 2021), YEY’s journaling approach, informed by the five emotional competences of the PEC model, facilitated a robust engagement with EI competences and coping strategies, evidenced in the resulting dataset. Using the PEC model as an analytical guide, the study examines how targeted competences, particularly emotion identification, expression, and regulation, are manifested across everyday situations in Lebanon. The present study thus aims to investigate how Lebanese adolescents engaged in an EI-based intervention to understand and enact EI competences in their daily lives through emotional journaling. In addition, reflective notes recorded by the Youth Leaders (YL) and Youth Facilitators (YF) who delivered the EI training sessions provided the research team with complementary observational insights into students’ emotional engagement during the training.

## Methods

### Study design

Our study used Colaizzi’s phenomenological method (Edward & Welch, 2011) in analyzing the emotional diaries that were part of the youth EI training that generated the data under review. Colaizzi’s theme-generating method of data analysis has been efficiently used in recent studies in the Lebanese context on the psychological impact of the August 4 explosion or of COVID-19 lockdown in Lebanon (El Hajj, 2022a; Fawaz et al., 2022, 2023). Moving from raw participant testimony to a structured understanding of the lived experience of crisis, the researchers in these studies applied Colaizzi’s rigorous framework to extract common emotional and psychological themes from those impacted by either the Beirut blast or the COVID-19 lockdowns.

As the above method focuses on the feelings and behaviors of participants in relation to lived triggers in their quotidian experiences, it is a cogent fit with the present study’s design, since it identifies shared patterns rather than individual characteristics in the study participants (Abu Shosha, 2012). While the Colaizzi approach does not quantify the prevalence of specific emotional experiences or behaviors, its modus operandi entails researcher immersion in the data, which are read and re-read (iteratively, as they were produced in the present study) to gain a holistic sense of participant experience. Later, theme clustering is coupled with exhaustive description and discussion, and enhanced with participant and/or facilitator validation. The YLs and YFs who led the training sessions verified that the final themes accurately represent the emotive realities they tackled in the classroom.

### Participants

Participants were Lebanese students enrolled in a semi-private high school in Lebanon. In the Lebanese educational context, semi-private schools are privately managed institutions, often operated by non-governmental or religious organizations, that receive support from the Lebanese Ministry of Education and Higher Education to provide subsidized education to students from low-income families. The selected school served a student population aligned to that targeted by the Yes to Emotions in Youth (YEY) program, namely adolescents from socioeconomically vulnerable backgrounds. The school was also selected on practical grounds, including its geographical accessibility to the research team and its willingness to host the intervention. Following approval from the school administration, students enrolled in the selected secondary classes (Grades 10–12) were invited to participate voluntarily in the program because they fell within the intervention's target age range of 15–17 years. The final sample comprised 145 students drawn from five classes. Of the participants, 59.8% identified as female, 35.4% as male, and 4.8% chose not to disclose their gender. During the sessions, a team of trained Youth Leaders (YLs), supported by Youth Facilitators (YFs), implemented the program.

### Outline of YEY implementation

The implementation of the YEY program took place once per week totaling five sessions, each lasting 45 minutes, which is the length of a full class period. Each of the five sessions focused on the five emotional competences of the Profile of Emotional Competence (Brasseur et al., 2013). These sessions were scheduled during regular school hours and replaced non-core subjects that were part of the school’s curriculum. The scheduling was coordinated closely with the school administrators with the goal of ensuring that the students experience minimal disruptions to their academic responsibilities. Initially, a focus group was conducted with one class of approximately 30 students. The focus group session consisted of an interactive Q&A sitting with topics about daily struggles, gender equality, bullying, and intrapersonal and interpersonal relationships. Following the focus group session, the five sessions of YEY were implemented with the aim of cultivating, embracing, and enhancing PEC’s five emotional competences (Brasseur et al., 2013). Using the information gathered from the focus group, the modules and presentations used in the program were adjusted, foregrounding the main interests and struggles discussed by the students during the focus group session. The evidence-based techniques used in the training included youth-led discussions, real-life examples, situational analyses, role play activities, and take-home exercises for each of the five modules.

### Data collection

At the end of each of the five sessions, participants were asked to take the emotional diaries home, complete them based on the prompts, and bring them to the following session. An average of four diaries were completed by each participant, one for each of the first four sessions. The final session that focused on the last emotional competence, the utilization of emotions, did not include a diary given that the intervention was terminated following the final module. Participants were asked to write short personal diaries after each session, reflecting on trigger events, such as incidents or situations that trigger emotional responses, and how they normally handle these situations in their daily lives. In particular, the emotional diaries followed the structure of the model of five emotional competences by Brasseur et al. (2013). Such a design allows focusing on individuals’ identification, understanding, expression, regulation and utilization of emotions as participants were going through the training sessions, while making discernible, through the diaries and their analysis, any potential shortcomings in the said individuals’ emotional regulation and/or coping mechanisms.

A sample prompt used for the EI diaries thanked participants for engaging with the relevant EI training session and invited the students to produce a short diary entry (300-600 words), where they would describe a situation first (what happened? what was the trigger?), then reflect on the emotion itself and emotion identification (what emotion did you experience? how did you know you were feeling that emotion in particular?—i.e., how did you identify that emotion in yourself?); emotional understanding (why do you think you felt that way? how did you perceive the situation?); emotion expression (how did you express, if you did, the emotion at that moment?—i.e., how did you show you were feeling that way?); emotion regulation and coping (what did you do while feeling that emotion? and afterwards?—e.g., something to feel better or improve the situation); and, finally, what was the outcome (what were the consequences? what happened afterwards?). These questions aimed at gathering data on the emotional competences from the PEC model (Brasseur et al., 2013). In the last question, participants reflected on what they learned from the experience.

In addition to keeping a record of the participant emotional diaries, the three Youth Leaders (YLs) and six Youth Facilitators (YFs) who delivered the training sessions were themselves requested to reflect and provide their observations, through post-training diary writing, on the emotional peaks and troughs of each session they led.

### Data analysis

The data was de-identified by R.C. by removing all personal information from the diaries and assigning each participant a numerical code during the study; a thematic analysis approach was adopted by the research team. The emotional diaries generated a substantial qualitative dataset, sampled in the subsequent tables in the study alongside notes excerpted from the YL and YF journals, and analyzed by S.EH. in light of a modified interpretation of Colaizzi’s seven-step method that accommodates thematic clustering and analysis (Edward & Welch, 2011). S.EH. read and reread each of the diaries to establish a general sense about their content; statements about the participants’ emotional responses in the trigger situations of their choice—their emotional identification and coping strategies—were extracted from the diaries. These “significant statements” were then used to create “formulated meanings” organized thereafter into themes and subthemes (Edward & Welch, 2011, 165).

Before the final themes and subthemes were discussed with MJ.SR., R.D., and M.EK.M., and tabled by S.EH. and R.C., all diary extracts were reviewed again by R.C. and the other YLs/YFs to make sure that the final results reflected the overall feel of the original diary entries. This step aligns with Edward and Welch’s call for versatility in applying and adapting “Colaizzi’s seven step analysis [to] offer researchers using Colaizzi’s method greater access to implicit and explicit meanings embedded in participant descriptions” (163). To ensure the trustworthiness and credibility of the qualitative analysis, and in following Colaizzi’s method, an iterative process of reviewing and clustering themes was used to maintain consistency between participants’ diaries and the resulting themes and subthemes. To provide additional insight and further validate the findings, the extracts from the participants’ diaries were tabled then analyzed alongside the extracts from the YFs’ and YLs’ diaries whose comments reflect on the training session after which each round of journaling was triggered.

### Researcher positionality and study ethics

As researchers involved in EI, psychosocial well-being, and adolescent development, the team approached this study with a commitment to youth empowerment. Some research team members had prior experience in implementing EI programs, which offered cultural insight but required continuous reflexivity to mitigate bias. To ensure analytic rigor, data coding was collaboratively reviewed and discussed, involving Youth Leaders and Facilitators, who validated emergent themes, confirming alignment with participant experiences. Decisions pertaining to the analyses were discussed among the members of the research team to promote consistency in the interpretation of the data. This reflective and participatory approach strengthened the credibility and contextual relevance of our findings. We also remained mindful of power dynamics between researchers and adolescent participants, striving to center youth voices throughout interpretation, and acknowledging the emotional weight of analyzing narratives grounded in distress and vulnerability.

Ethical approval for the study design and data collection was in accordance with the Declaration of Helsinki and was secured from the pertinent Institutional Review Board (tracking number LAU.SAS.MJ1.28), approved too by the Lebanese Ministry of Education and Higher Education. Informed consent forms were obtained and signed from parents whereas oral assent was obtained from the students. The consent forms tackled both participation in the EI training sessions and the use of students’ emotional diaries for research purposes. Participants were handed a copy of the consent form and were made aware that their participation in the training was voluntary and that they had recourse to psychological support through a trauma specialist who was available during the training and afterwards, should they experience any form of emotional distress or duress during the training.

## Results

The thematic analysis yielded themes illustrating how participants expressed and applied the EI competences covered by the YEY intervention within their everyday experiences. Given the short-term nature of the program, the analysis was not intended to assess developmental change, but rather to examine how these competences were articulated and enacted through participants’ journals. Nonetheless, as the intervention progressed, prompting participants to ponder several aspects of their lives, they provided unsolicited verbal feedback regarding their EI learning experience, recorded thereafter in the diaries of the YLs/YFs who delivered the training: “A few came to thank us and stated that they were applying the EI strategies we taught and found them very helpful […]” (YF1). Although some students were initially resistant to the Youth Leaders’ and Facilitators’ EI-based approach, they gradually recognized the core purpose of emotional expression and the broader significance of emotional intelligence (YF2).

In line with Colaizzi’s methodology, the research team identified, described, and discussed four overarching themes reflecting the participants’ negotiation of EI competences in their diaries: (One) Emotional identification: unpleasant emotions and triggers; (Two) Emotional identification: pleasant emotions and triggers; (Three) Coping and emotional regulation: maladaptive practices; and (Four) Coping and emotional regulation: adaptive practices.

Emotional identification in the findings features emphasis on both unpleasant and pleasant emotions and their concomitant triggers. Similarly, the theme of coping and emotional regulation diverges into destructive or maladaptive, and constructive or adaptive strategies or practices of processing emotions, especially in response to duress, adversity, or stressful events. Exemplar excerpts for each of the four themes are displayed in Tables A1 to A4 in the appendix section. Considering that the selected student quotes overlap between the two discernible subthemes (triggers + emotions) for themes (One) and (Two), the identified emotions therein are highlighted in Tables A1 and A2 for ease of access.

### Theme One: emotional identification: unpleasant emotions and triggers (Table A1)

Summary of reported emotions: sadness, stress, anxiety, anger, hate, fear, impulsivity, confusion, exhaustion, and guilt. The participants divulged four sets of stress factors, each triggering a range of unpleasant emotions—*how* they recognized these emotions was also revealed in several entries. A few youths cited illness in the family or death of a loved one as triggers for sadness, fear, anxiety, anger, and impulsive behavior (AG1012: “I feel scared seeing my dad at his worst lying on his deathbed. It was hard saying goodbye to the one you love after being happy and having good times around him”). In addition to bereavement and health concerns, school stressors elicited anxiety, loneliness, and sadness related to students worrying about their academic performance.

The YLs and YFs remarked on the occasional tension among the students during the training sessions, sometimes due to uncouth behavior, such as comments about appearance that were meant as jokes but actually sparked emotional pain, or interrupting a student who is expressing how s/he feels by “making pejorative comments or joking around” (YF2). Along these lines, school misbehavior, of unspecified nature in the diaries, was cause in turn for feelings of guilt, regret, and shame, and the inability to process these feelings (ON2004: “I was experiencing much guilt because of what I did at school. I couldn’t look my parents in the eyes [...]. I avoided talking to [them] and didn’t mention school”).

A YF noted in their diary that many students struggled with interpersonal relationships, including at times with others in class: “One student told another in a role play activity: ‘It’s your duty as a girl to arrange the dining table, not my duty’” (YF1). Indeed, turbulent interpersonal relationships, tabled as relationships in/of conflict, were behind much of the distress and listlessness described in the diaries. Confusion, anger, sadness, and guilt were fomented by friendships strained by disagreements, misunderstandings, fights, and arguments. The participants recognized these emotions because they triggered “excessive thinking” about the conflict, as well as an inability to smile or laugh, coupled at times with impulsive behavior (GM0112: “I fought with my best friend. I felt sad, and because of my excessive thinking I knew I was sad because it showed on my face”).

Moreover, participants reported numerous instances of parent-child discord as catalysts for distressing emotions and dysfunctional and/or halted communication with their parents. These examples included a lack of mutual respect, missing school because of family conflicts, and a father’s inability to understand the implications of his unwillingness to settle unpaid school tuition fees. Anger, anxiety, and sadness, even helplessness and desperation, were the emotions identified by and in students’ diaries because of the frustration implicit to tone-deaf, dysfunctional parent-child relationships. As the recognition factors behind these emotions, students’ diaries specified sleeping problems, crying episodes, disrupted routines, and accelerating heartbeats. Indeed, several students discerned their feelings somatically, identifying them through the physical changes they reported in their diaries:

-I recognize my feelings through a change in my body language, like whenever I’m feeling nervous or anxious, I start stuttering and shaking my legs, which I did a lot. (MC0605)

-I had my hand in my mouth and my leg was shaking; I could tell the constant thinking was making me anxious. (AA0716)

Finally, a general and inchoate state of malaise/anxiety, mostly without a cogent or apparent trigger, was dominated by incessant distressing thinking or disruptive rumination, as well as a persistent sense of insecurity. While the former triggered anxiety, exhaustion, and feelings of being judged—unpleasant emotions hampering the efficient functioning of the concerned participants in their respective everyday lives—the latter engendered, inter alia, strong emotions of sadness, anxiety, nervousness, and insecurity.

### Theme two: emotional identification: pleasant emotions and triggers (Table A2)

Summary of reported emotions: happiness, relief, comfort, success, surprise, excitement, pride, and gratitude. The emotions identified by the participants in their diaries were triggered by a range of activities and pursuits, all of which are subsumed entirely under autonomous behaviors. The latter term refers in psychology to actions based on independent decision-making, unaffected by peer, family, or social pressures. A person’s decision and its corresponding action(s) are thus motivated by personal values and goals instead of external influences (Bergamin et al., 2022). Engagement in autonomous behavior, as per students’ input, resulted in pleasant feelings of satisfaction, fulfillment, and general well-being.

Autonomous behaviors described in the findings featured a slew of sports and hobbies, such as basketball, football, drawing, music, camping, shopping, and cars. For other participants, the self-chosen activities and pursuits that triggered pleasant emotions included undertaking new activities, such as training for personal or professional development, familiar activities, such as churchgoing and quality time with loved ones, or doing something for others. For at least one student, autonomy came in the form of being recognized by their parent as an equal—as a friend, not a child, who would be respected and listened to, and therefore empowered. Participants linked their experience and subsequent identification of pleasant emotions to improved energy levels, involuntary smiling, and increased heart rates:

-We went camping with the altar servers in my parish. I felt an extraordinary amount of happiness. I knew I was happy because I was very energetic, and because others noticed and told me so. (SM271)

-I went to see someone dear to me that I missed. I felt happy and comfortable; I knew that from the smile that appeared on my face and my heart started beating quickly from all the happiness I was feeling. […]. I didn’t want to leave. (GM0112)

The tabled YL/YF diaries, post-training, similarly confirm the participants’ need—whether they are in class or at home—for agency, self-assertion, and voice, and their concomitant possibility of autonomous choices and behaviors. There was active engagement in the EI activities presented, since “participants want[ed] the space to speak and be heard, and whenever they talked and realized that we were listening to them, they were happy” (YL2).

### Theme three: emotional regulation and coping strategies/maladaptive practices (Table A3)

Different practices that cohere with stress management processes, both healthy/adaptive and unhealthy/maladaptive, were reported by the youth participants. Unhealthy coping and emotional regulation strategies were informed by dysfunctional avoidance, emotional non-expression, and unregulated aggression. Avoidance behaviors in which the cause of stress or trauma is shelved rather than addressed, and are therefore closely linked with “affect dysregulation” or maladaptive coping (Briere et al., 2010), appear in the diaries as strained parent-child dynamics shaping the (mis) communication impasses pervading various participant experiences.

The most encountered vicissitudes in emotional and personal well-being, in relation to troubled relationships, were predicated on discordant parent-child dynamics. Several participants also expressed dissatisfaction with their schoolteachers’ EI competences. The YLs and YFs shared that, in terms of conducive emotional expression or communication, these students found that often “their teachers communicate with them maladaptively” (YL1). Students’ troubled relationships led to a status quo of self-isolation symptomatic of dysfunctional avoidance, and accompanied in some cases with impulsive behavior, such as absconding from home, as well as substance abuse, such as escapist intoxication. ES1008 is a case in point. “I usually drink [alcohol] to deal with any situation that I can’t control,” the student writes, “and this relaxes me.”

In addition, two key strategies aligning with maladaptive coping or affect dysregulation were discerned in the participants’ input: emotional non-expression and unregulated aggression. Whether in denying or suppressing the expression of emotion, emotional non-expression recurs in the diaries, sublimated in at least two entries into emotional eating. Abuse is further evident in the reported experiences both in its verbal and physical forms, toward self and others, and is corroborated in the observations of the YLs/YFs conducting the EI training. They write that, some youths, in believing that “they shouldn’t let anyone eat their head” [overpower them], consider responding to abuse, in whatever manner, with similar abuse, a viable coping strategy (YF2). In this respect, hitting and biting others (and themselves) was raised in the class discussions and youth diaries alike:

-I did something that I haven’t done in a long time. I started hurting and cutting myself again. (CC1703)

-When we fight, I yell at and punch everything. (GN1507)

### Theme four: emotion regulation and coping strategies/adaptive practices (Table A4)

The study findings include, too, healthy or adaptive practices of coping or emotion regulation, all exclusively in response to stress triggers or daily conundrums. The first of these is functional confrontation, whereby the participant would, for example, approach a friend about the pertinent problem, to discuss and potentially resolve it. Others reported tackling their stressors head-on, booking doctors’ appointments to resolve health-related anxieties, or deciding to change their studying habits to optimize their academic performance and related well-being.

Second, reorienting attention through a cognitive reframing or reevaluation of a potentially stressful situation or setup was reported by some students who endeavored to rethink their approach to, and where applicable anticipation of, stress and its triggers. Deciding to apply diligence in their pursuit of a better academic performance, or of simply a “freer” mindset unlabored with anticipation or projection biases, meant that previously stressful situations, such as braving family weekends or studying for exams, became easier to manage. In this manner, a change in perception, namely a willful cognitive shift, is common to the participants’ deployment of this strategy, not only regulating the unpleasant emotion associated with the stressor they described but also shifting it altogether (MC0605: “Today I started seeing things in a different perspective and my body was less stressed. I think I started to feel like this because I changed the way I see things”).

Reorienting attention was also resorted to by some participants in the form of functional distraction. The diaries noted a range of activities, namely drawing, listening to music, reading, downtime with friends, and watching television, that also facilitated leeway for some participants to dampen and temporarily override intense emotions, before mustering the energy and determination required to grapple with their stressors (JS1005: “I went to my room and drew to forget”; CS0104: “At the end of the stressful day, I decided to go to the *kermesse* [fair] to change my thoughts and mood”).

Physio-relaxing and other self-care techniques were revealed by the students as effective choices for affect regulation, whether through physical activity, breathing exercises, and/or prayer (SM271: “I prayed a lot to the Virgin Mary; what happened after was that I was shown a great sign and I stuck to my call even further and got out of the confusion”; SR1603: “I controlled [my] fear with prayer”). Crying, too, appears in the diaries as a means of self-soothing or tension release. After a mindfulness exercise, for example, two girls were crying; “it had proved cathartic to them,” wrote a youth leader (YL1).

Finally, emotional support, often through the cultivation of edifying friendships, as well as logging and pondering difficult emotions through diary writing, was among the emotion management resources in the present findings. In turn, the YL/YF diaries noted students sharing with their trainers that diary writing “helped them discover themselves and learn about issues they had to work on” (YF2).

## Discussion

Although the results appear to be attuned to the PEC components, this alignment is intentional, both methodologically and conceptually. The study’s diary structure, in subsuming the five emotional competences of the PEC model, encouraged critical analysis of emotional competences and deeper engagement with coping strategies—the outcome being the layers of data, responses, and triggers, discernible in the student journals and validated initially through the reflective observations in the YL/YF diaries. Colaizzi’s phenomenological method allowed, as above, for a detailed pre-discussion description of themes that only a robust dataset makes possible, and which in this study was facilitated by linking the diary structure to the PEC model.

The described findings offer insight into different aspects of EI, namely emotional identification and expression, and affect regulation/dysregulation, as experienced by adolescent youths living in today’s Lebanon. These findings provide valuable insights into the emotional competences of Lebanese youth, building upon existing trait EI research and offering a nuanced perspective on the five emotional competences model proposed by Brasseur et al. (2013). By analyzing emotional diaries through Colaizzi’s method, this study offers a rich, contextual understanding of how adolescents in Lebanon engage with their emotions amidst ongoing societal challenges.

For many students, emotion recognition through crying and increased heartbeats is similar across adaptive and maladaptive emotion regulation, nodding to previous research examining these factors as physiological responses immaterial to cultural context (Critchley et al., 2004; Gračanin et al., 2014). Similarly, the articulations of EI among Lebanese youth reasonably suggest that reported frustrations stem chiefly from developmental stage, albeit potentially amplified by the patriarchal cultural environment. In some of the reported findings, such as emotional non-expression and its consequences, the local setting might trigger, worsen, or preclude EI coping and learning, but context alone may not exclusively define the results in youth research in Lebanon.

This versatile understanding aligns with broader qualitative studies on EI in non-Lebanese youth populations (Crockett et al. 2022; Van den Bos et al., 2014), and speaks to previous research, which finds that age-related developmental shifts—due to neurobiological maturation, hormonal changes during puberty, and evolving cognitive processes—significantly influence teenage mental health and stress responses, often in conjunction with their social context (Casey et al., 2008; Dahl & Gunnar, 2009; Steinberg, 2005; Van den Bos et al., 2014).

### Coping versus stress-unrelated emotion regulation

While the sampled population generally demonstrates some level of proficiency in identifying both pleasant and unpleasant emotions and their triggers, many of the youth participants struggled with processing these emotions adaptively. This finding aligns with previous research on emotion regulation challenges among youth in Lebanon, exposed to ongoing sociopolitical stressors, thereby exhibiting difficulties in adaptive emotion regulation (Baroud et al., 2024). Hence, even as the findings from the present study are potentially consolidated by context, they must be interpreted considering broader theoretical distinctions between coping and emotion (dys)regulation. Emotion regulation, regardless of developmental stage, involves deliberate or automatic processes aimed at modulating emotions across a broad spectrum of situations—*not only in response to stress*, but also during normative and even pleasant circumstances (Compas et al., 2001; Folkman & Moskowitz, 2004). For example, regulating the urge to stay up late on the eve of a holiday, or suppressing anger in response to a peer’s disingenuous boasting, are acts of emotion regulation that may not be motivated by stress per se.

By contrast, the adolescents’ emotional narratives in this study appeared to reflect coping strategies, which are more narrowly defined as responses to stressful situations. This is consistent with Compas et al. (2014) conceptualization of coping as a behavioral pattern activated by perceived stressors, which seems to align with the way emotion-related challenges are described by the participants in both their diaries and classroom discussions. This distinction between coping and emotion regulation gains further meaning when contextualized within the concept of Lebano-pathography (El Hajj, 2021b, 2023), a term capturing the chronic socio-political, personal, and economic hardship that permeates daily life in Lebanon. These structural adversities may have normalized a “survival mode” orientation among youth and adults alike, potentially narrowing adolescents’ awareness or acknowledgment of larger systemic stressors. Instead, their emotional energy may be concentrated on navigating proximal, developmentally salient concerns. This phenomenon underscores the adaptive value, but also the potential limitations, of coping strategies when emotional resources are continuously taxed by protracted adversity.

### Emotional non-expression, dysregulation, and patriarchy

Even as most diaries in our study eschew explicit statement of the Lebanese milieu, exemplar findings or excerpts from students’ diaries can only accrue meaning or value beyond individual experience, once examined in light of the context that shaped them. One student telling another, for example, that arranging a dining table is a woman’s duty bears implications beyond the scope of a role-play activity. Similarly revelatory may be the youth references to prayer as a coping mechanism. In focusing on individual endurance, prayer, as Tomalin (2013) has argued, is not only socially condoned, but also helps alleviate the somatic anxiety of inhabiting an oppressive patriarchy or a corrupt social fabric.

Context is therefore crucial in understanding the study’s tabled inputs, which are in line with other types of diary studies or life writing explorations in biography and ethnography, in which youth perceptions of well-being in relation to home are complicated by a systemic patriarchal status quo, dominating all social and political aspects of life in Lebanon (Khalaf, 2009, 2014; El Hajj, 2022b, 2026). As these studies concur, emotional expression is often actively clamped down in patriarchal contexts, particularly for men who are pressed to keep, in private as in public, patriarchy’s “stiff upper lip” (Dodsworth, 2017, 9), and for women who are encouraged to pray (Tomalin, 2013).

This cultivated ethos of emotion non-expression, which translated in the early days of the EI training into a resistance to the emotional expression activities conducted by the YLs/YFs, takes a toll on everyone, men as well as women. Since “almost all of the students discussed […] their relationships with their parents that seemed dysfunctional,” the YFs/YLs attested to a pressing need to “fight stigma against emotional expression,” to better negotiate parental relationships that have suffered as a result of maladaptive emotional communication. The less skilled individuals remain at expressing and thereafter regulating emotions, the more likely they will use inappropriate coping strategies and the higher the risk of harm to their well-being or quality of life through stress or unpleasant emotions (Baroud et al., 2024). Socially ingrained emotional non-expression may thus explain the emotional challenges manifest in our findings, in relation to not only the concerned participants, but also to key stakeholders in their surroundings, namely their parents at home and teachers at school.

In fact, in the present study, a stance regarding emotional expression shared by several students, boys and girls, in their diaries and in class discussions, was that “there’s no point in expressing emotions, and this would only show the other person that we are ‘weak’.” Idioms of aggression infiltrate daily life in Lebanon, both in rhetoric and in practice, impacting, as some youth diaries imply, people’s willingness to actively engage in EI processes, such as self-reflection and emotional expression. Thus, students’ fear of being perceived as weak or the exhortation to “not let their heads be eaten” can be viscerally linked to the power dynamics that undergird personal relationships in a country governed by patriarchy and still recovering from civil war (El Hajj, 2018; Khalaf, 2014), and, as of this submission, presently undergoing war.

Denial, emotional suppression, and dysfunctional avoidance are, moreover, different ways of emotional non-expression compromising participants’ health, whether physical (Gross, 2002) or mental (Nelis et al., 2009). These findings are worrying in that many of the students’ diaries show an alarming recourse to destructive emotional regulation mechanisms, including physical violence to self and others, verbal abuse, absconding from home, and alcoholism. To this effect, a YF noted that even when students in a class discussion engaged the topic of alcohol addiction, “it was clear that a lot of them did not understand its severity, or the risks of using alcohol as a coping method” (YF1). There is therefore a sharp need for developing and mastering more adaptive coping tools responding to stressful triggers, and in doing so substituting for the maladaptive strategies that have cast a pall on the youth participants’ quotidian encounters with conflict.

### Autonomous behaviors and emotion regulation

Notwithstanding the extent of affect dysregulation and references to unpleasant emotions in the study, it is important to also note that some of the participants appear to cope well with their stress triggers and their resultant pleasant emotions, in terms of thoughts, behaviors, and social relationships (Fredrickson & Branigan, 2005). What functional confrontation, reorienting attention, and physio-relaxing techniques—the adaptive emotion regulation strategies identified in students’ input—have in common is, on a very basic level, a joint acknowledgment of the stress factor and an individual willingness to take action to improve or deescalate pugnacious situations. Although these strategies are not new per se, what is remarkable is that all the culled triggers for pleasant emotions can be interpreted as autonomous behaviors enacted by the participants themselves.

In psychology, autonomy is viewed as essential to individual well-being, motivation, and psychological health. Individuals who are able to engage in autonomous behavior are more likely to perform well at school, and later at work, and to generally feel happier and achieve better self-esteem than their counterparts whose decisions others control (Bergamin et al., 2022; Kukita et al., 2020; Legault, 2016). Along these lines, a YF observed that one student considered her mother as “emotionally intelligent because she treats me like her friend, not as her daughter” (YF3). In listening to youth, and giving them the space to thrive, learn, and make independent decisions, they may fully tap into and benefit from the pleasant emotions spawned by autonomous behaviors and their sense of mastery.

### Research and practical implications

The study’s focus on emotional diaries, as both an intervention tool and a data collection method, provides a unique perspective on the development of emotional competences. By encouraging participants to reflect on their emotional experiences, the study not only gathered data but also potentially enhanced participants’ emotional self-awareness—a key component of trait EI (Petrides et al., 2016). This approach aligns with a growing body of research supporting the use of structured journaling as a facilitator for improved emotional clarity and psychological well-being (Brans et al., 2013; Pekaar et al., 2018).

The study makes a significant contribution to trait EI research by adopting a qualitative approach in a context where quantitative methods have predominated. While previous studies in Lebanon have established connections between trait EI and mental health (Sanchez-Ruiz et al., 2021; Zeeni et al., 2018), this qualitative exploration provides deeper insights into the lived emotional experiences of Lebanese youth. The thematic analysis of emotional diaries offers a more nuanced understanding of how trait EI manifests in daily life, particularly in a challenging sociopolitical environment. The concomitant findings on coping strategies and emotional (dys)regulation further enrich our understanding of trait EI in high-adversity contexts. Participants described the use of maladaptive coping mechanisms, such as emotional suppression or disengagement, as well as a perceived lack of emotional competence among teachers. These insights suggest that prolonged exposure to sociopolitical instability may impair the development or expression of adaptive emotional strategies. Such observations complement prior quantitative findings linking higher trait EI to more effective coping mechanisms (Mikolajczak & Luminet, 2008; Zeidner et al., 2004), and underscore the importance of nurturing emotional skills across both students and educators.

By examining how Lebanese youth navigate adversity using the YEY program and qualitative methods, the study also addresses a critical gap in EI research within the Arab Middle East (Nassar et al., 2023). The findings offer culturally grounded insights with practical implications for the design of social-emotional learning (SEL) initiatives. They highlight the urgent need for targeted interventions that support emotion regulation and emotional literacy in adolescents facing chronic stress and uncertainty.

### Limitations and recommendations for future research

Although this paper is one of the first qualitative studies exploring EI in Lebanese youth via emotional diaries, several limitations can be addressed in future research. First, due to a lack of consistency in filling out the journals, the available data did not allow for comparing the changes in emotional competences before, during, and after the EI training program. Future studies could use post-implementation journals to assess the usefulness of the program in the students’ emotional competence learning process, together with any post-assessment quantitative methods. In addition, the levels of narrative skill and self-disclosure may have varied across participants’ diaries, potentially influencing data variability. Future studies could benefit from a larger and more diverse sample of participants to fill out the journals. Despite these limitations, through using emotional diaries as both an intervention and analytic tool, this study highlights the methodological potential of reflective writing in adolescent mental health research.

## Conclusion

Knowing that “coping and emotion regulation are distinct but closely related constructs” (Compas et al., 2014, 71), the sampled population, and by extension other youths living in Lebanon today, need to develop aspects of emotional regulation not only strictly tailored to coping in episodes of stress or duress. This study reveals that while the sampled population is, in general, attuned with, and can clearly identify, emotions and their pertinent triggers, many of the youth participants struggled with processing these emotions adaptively, and largely did not engage with stress-unrelated emotion regulation. Therefore, particularly in contexts of turmoil, the need to efficiently grapple with this crucial EI competence, emotional regulation, highlights the importance of such training in EI at a time when youth mental health issues are elided in Lebanon.

An important finding of the study remains the nature of the stressors reported by participants. Despite the unprecedented and ongoing crises in Lebanon, including political instability, economic collapse, and infrastructural failures, adolescents in our sample did not, as mentioned, foreground these macro-level adversities in their emotional diaries. Instead, the stressors they described were more typical of developmental concerns commonly observed across adolescent populations globally, such as parental discord, academic pressures, and peer-related conflicts. This suggests that, even in highly distressed national contexts, adolescents’ grievances may be consonant with the immediate interpersonal and developmental challenges of their age group. Future research could therefore examine whether similar patterns emerge in other cultural contexts and compare adolescent populations across settings to better understand which findings reflect developmental processes and which could be context-specific.

Finally, findings from this study underscore the necessity of integrating emotion regulation training into youth mental health programs, particularly in crisis contexts where stress is normalized. The YEY intervention demonstrates the value of culturally grounded, youth-led approaches. Future research should evaluate long-term impacts and expand to more diverse school settings, contributing to evidence-based policy in the Middle East and beyond.

## Data Availability

The data that support the findings of this study are available from the corresponding author, M.EK.M., upon reasonable request.

## Appendix

**Table A1.** Theme one—emotional identification: unpleasant emotions and triggers.

Triggers	Emotive quotes from students		Quotes from YLs and YFs
**I. Illness and Death**			
(1) Bereavement	-I feel **scared** seeing my dad in his worst lying on his deathbed. It was hard saying goodbye to the one you love after being happy and having good times around him. (AG1012) -The death of my uncle caused me much **sadness** and **stress**, because I love him and it was a big shock for me. I cried so much and I tried to sleep, but I couldn’t make my brain stop thinking. (AN0808)		
(2) Health concerns	-I felt **scared** because I was in pain and had to get it checked by a doctor. I was a little scared to hear the results, but I controlled this fear with prayer. While I was scared, I was always in my room, **worried**, thinking too much about it. (SR1603) -I made a hard decision to follow a diet and this made me feel **angry** and **impulsive**. (TP1311) -My grandpa got into the hospital. I was really **scared**, **anxious**, and **sad**. I was afraid that something might happen to him. I started overthinking all the scenarios that might happen. (MC0605)		
**II. School Stressors**			
(1) Academic Performance	- I returned from school so tired from my teachers and my books; I felt **lonely** and **sad**. I wasn’t feeling enjoyment and enjoyment wasn’t feeling me: I was seeing black everywhere in the sky, in my bedroom, in my parents’ faces and it was completely terrible! (ML1812) - I have felt a lot of **stress** because exams are nearing. (CG0207)	-The students enjoyed the role play exercises pertaining to emotional expression. One of the things they shared, for instance, was that sometimes their teachers communicate with them maladaptively. (YL1)	
(2) School Misbehavior	-I was experiencing much **guilt** because of what I did at school. I couldn’t look my parents in the eyes and I also felt **regret** and **shame**. I avoided talking to my parents and didn’t mention school. (ON2004)	-There seemed to be tension among the students in some of the training sessions. One student specifically caught [my] attention because it seemed like he was actually opening up, but students who were sitting down were making pejorative comments and joking around, which made him visibly uncomfortable, so the role play was cut short. (YF2) - One girl expressed that one of her classmates hurts her when he comments on her weight (he said it as a joke and did not know that this was hurting her), so that opened a discussion on the importance of adaptive and respectful communication and emotional expression. (YF1)	
**III. Relationships in/of Conflict**			
(1) Strained friendships	-I feel a disturbance in my relationship with my best friends. I feel **confused**, **angry**, and also **sad**. […] Confusion is a result of me not knowing why I feel that way. (ON2004) -After we argued, I couldn’t spend time with my friend and that made me feel **unhappy** because I love her. (TP1311) -I fought with my best friend. I felt **sad**, and because of my excessive thinking I knew I was sad because it showed on my face. I wouldn’t manage to smile or laugh about anything. (GM0112) - A disagreement with a friend made me feel **guilty**, **sad**, and **angry** at myself. I was thinking a lot before sleeping and I was acting and speaking impulsively. (TP1311) -This week a misunderstanding happened between a close friend and me. I felt **angry**. I knew it was anger through my reaction to the subject and because I was constantly thinking about what happened. (ES1008)	- One student told another in a role play activity: “It’s your duty as a girl to arrange the dining table, not my duty.” Based on after-session discussions, I noticed that many students struggled with interpersonal relationships, with friends, siblings, and others. (YF1)	
(2) Parent-child discord	-[…] my mood was trash, so I got **angry**. She was saying “respect me…” well you respect me first how about that. (CS0104) -It’s not that I hate home, but I had school, and, because of the problems at home, this was the first time I get absent since KG2. I was **anxious** and **angry** about it. I had never missed a class and I rank first in my class! (ML1812) -All I could feel was **sadness** and **anger** […]. I didn’t say anything and walked out from the house. (AN0808) -My parents took my phone from me; it has been a long time, and they said if I get good grades, I will get it back. I got so **angry** with them at first but then with some time I calmed down and I now I feel really **sad** and **desperate**. (CC1703) -I felt **stressed** because my parents put the blame on me in every situation, and I get **angry**. (LB0803) -I was feeling very **sad** and **angry**, because I chose as an outfit for my sister’s prom something rather “masculine” and I liked it very much, but later that night my father told me you can’t wear that. I knew I was feeling this way because I didn’t sleep well that night. (CR0310) -The problem that I’m facing is my parents’ inability to understand me. And that’s what makes me feel very sad and constantly **angry** now. I realized that I’m **sad** because of my constant crying and my inability to spend a good day. (CS2412) -I fight with my parents over […] and I feel **stressed** and **worried**, and **angry**. I isolate myself and go to my room and don’t feel like talking to anyone. (RY2007) -Two days ago, I called my dad whom I haven’t seen in a long time to tell him about the importance of paying my tuition, but he didn’t care about it and refused to help. At that moment, I felt an indescribable **anger** and my blood boiled. (RR1401) -I experienced a misunderstanding with the family, a small conflict occurred, and I felt so **angry**. I couldn’t express how I was feeling because when I get angry at something, I feel like crying and get bothered and I sit alone so that my anger dissolves. (LZ2807) -I felt **sad** because of family problems caused by the local elections. I recognized this feeling because I don’t like yelling and my heart was beating quickly. I was in my room alone. (JS1005) -I feel extreme **sadness** and **anger** due to my family problems. Words cannot describe the emotions I am feeling. (RR1401)	-Some students discussed issues that they face at their houses with their parents and expressed their inability to properly communicate with their parents. (YL2) -It was in the session on emotional expression where the first activity triggered someone, whereby the prompt solicited reactions to a mother assigning the house chores to her daughter, and that made things a little uncomfortable for the girls in the classroom. (YF1) -The students also imitated their parents during role play, and you could see how disturbing it was to them to revisit the parent-child dynamic. (YF3) -The role play activity reflected what some of the students are going through in their private lives, specifically when one individual answered, “You’re my mom, do you even ask about me?” Real-life situations/emotions were portrayed during the role play. (YF4) -Students implicitly revealed being neglected by their parents; they talk with their parents in a dysfunctional and maladaptive manner, never reaching common ground, which was portrayed in the role play and other training activities. (YL1)	
**IV. General distress/anxiety**			
(1) Disruptive rumination	-I feel **exhausted**. That’s from thinking too much about every little thing in my life. (MR2508) -I wanted to date a girl I previously met at a birthday party. […] I was sitting, staring at the wall and I had my hand in my mouth and my leg was shaking; I could tell the constant thinking was making me **anxious**; a few minutes passed while I was sitting this way. I had a long talk with myself in my head, which helped me gain back my confidence. (AA0716) - I always think that no one accepts me and I have that weird feeling that I am always **being judged**. (CS0106)	-The boy that normally has a laid-back exterior, even when speaking in front of the whole class, seemed quite uncomfortable talking about his emotions with the YF (evident through the student’s body language). (YL1) -The students were very resistant to suggestions for the first activity on self-expression. They would say: “*I don’t want to express how I feel*; it’s true that sometimes no one understands me.” (YF2)	
(2) Insecurity	-I feel unloved and **unhappy** even with friends. (CS0104) -Most of the time I feel **depressed** out of nowhere and this happens a lot and I don’t know why, and it could be thinking of dark things that make me even more **depressed** and **insecure**. (AM54) -I was feeling so **anxious** about basically everything like my grades and my general wellbeing. I was also so afraid that in every situation something bad was going to happen. I recognize my feelings through a change in my body language, like whenever I’m feeling **nervous** or **anxious**, I start stuttering and shaking my legs which I did a lot. (MC0605)	-One student shared her opinion when a small discussion was taking place; she indicated that she views the society in Lebanon as one that discourages the expression of emotions and that she finds this harmful. (YF1)	

**Table A2.** Theme two—emotional identification: pleasant emotions and triggers.

Triggers	Emotive quotes from students	Quotes from YLs and YFs
**I. Autonomous Behaviors**		
(1) Sports & Hobbies	-My biggest **relief** and **happiness** is when I go to school to play basketball, my favorite activity. (TP1311) -I felt really **happy** because my workouts, school, football training, all went really well. I felt good on the inside, stress-free. (MC2608) -I sat down listening to calm music and I started drawing. I knew that a smile would appear on my face when I finished drawing; I felt **relieved** and **happy**. (GM0112) -I heard a car while it was popping, and I felt **joy** and **happiness**. And I knew I felt that way because of my smile and the good energy running through me. I think I felt this way because I love cars. They are my hobby. (GJ1801)	-There was interest and active engagement in the activities presented. (YL1) - The youth participants want the space to speak and be heard. And whenever they talked and realized that we were listening to them, they were happy. (YL2)
(2) Other self-chosen activities and pursuits	-We went camping with the altar servers in my parish. I felt an extraordinary amount of **happiness**. I knew I was happy because I was very energetic, and because others noticed and told me so. I think I felt so hyper because, deep down, I was happy. (SM271) -Friday I went to the jewelry shop to buy a necklace for my mom’s birthday so I was **happy** knowing she would be too. (RR1909) -I took the decision and went to the gym. I left it feeling **successful** and **happy**, because I accomplished something I had never done before. (AN0808) -Buying the dress I wanted made me feel **happy** because I like getting things. (SF0806) -I went to church. I feel **happy** and **comforted** there. I knew I would feel this way, with the smiles and good vibes. (GJ1801) -I decided to sign up for sessions on leadership and other soft skills. I loved these. I feel so **proud** of myself. (CS0104) -Last Tuesday, I spent time talking to a girl I’m close to at school, which gave me a feeling of **happiness**. (EL1707) -I joined my pals to celebrate our friend’s birthday. I was smiling and **comfortable** around them and we were laughing together. (JS1005) -I went to see someone dear to me that I missed. I felt **happy** and **comfortable**; I knew that from the smile that appeared on my face and my heart started beating quickly from all the **happiness** I was feeling. […]. I didn’t want to leave. (GM0112) -My uncle flew in from Zambia, and I arranged to see him. I felt so **happy** and **excited** because I had missed him so much. (GD2401) - In the past week I’ve been feeling good about my body, my life and the way I am starting to understand things. I knew I was **happy** with my decisions because I couldn’t stop smiling. I felt **grateful**, relaxed, and **happy**—I felt that I should just think about **happiness**. (MC0605) -Studying hard pays off. Good grades at school give me joy. I feel **pride** and **happiness** on the inside. (CS0606)	-My mom is emotionally intelligent because she treats me like her friend, not as her daughter. She listens to me. (YF3) -The students who did the gratitude part of the homework gave a bit of feedback by saying it allowed them to dig deep and think about several aspects of their lives. (YF4) -A few came to thank us and stated that they were applying the EI strategies we taught and found them very helpful, and many came to see us, to share personal questions and worries. (YF1) -Several students who were at first disagreeing with almost every idea/concept discussed by the Youth Leader started to better understand the goal behind understanding and expressing emotions, and the importance of being emotionally intelligent overall. (YF2)

**Table A3.** Theme three—emotional regulation and coping/maladaptive practices.

Subthemes	Quotes from students	Quotes from YLs and YFs
**I. Dysfunctional Avoidance**		
(1) Communication Stalemates	-I avoided talking to my parents and didn’t mention school. (ON2004) -My parents were so angry when they found out what we had done, so we ran away to my cousin’s to avoid dealing with them and the next day to change our mood, we went to the mall and we teased people. (GT137)	-One student said she couldn't do the mindfulness exercise. When [a YL] talked to her privately, she said that she had too many negative emotions and she feared that this activity would make her relive them; she even didn't want to be in class, so we agreed that she could wait out on this discussion. (YF4) -The idea of adaptive expression of emotions, let alone adaptive emotional regulation, didn't seem realistic to the participants, especially when it came to healthy communication with their parents. They felt that it was impossible for them to communicate with their parents in an adaptive way that follows the four key points of adaptive expression. (YL2)
(2) Impulsive Behavior	-My cousin and I went to an abandoned house the day we fought with our parents; it was creepy, but we had fun. (GT326) -I cut my hair myself when feeling overwhelmed. (ZB1705) - I put action to word and ran away from home. (MX519)	
(3) Substance Abuse	-I usually drink [alcohol] to deal with any situation that I can’t control and this relaxes me. (ES1008)	-A debate about addiction, especially alcohol addiction, started in class among students; although it was interesting to listen to their thoughts, it became clear that a lot of them did not understand the severity of alcoholism, or the risks of using alcohol as a coping method. (YF1)
(4) Self-isolation	-I pushed everyone away. (BF9076) -When I’m upset, I distance myself from my closest people and loved ones until I feel good again. (MT1707) -I isolated myself from the world and my friends. (AA0809) -I isolate myself and go to my room and don’t feel like talking to anyone. (RY2007)	
**II. Emotional Non-Expression**		
(1) Denial	-But whenever something bad happens like just before now, I would simply convince myself it didn’t happen even if it’s still bothering me on the inside. (GN1611)	
(2) Emotional suppression	-I tend to bottle my feelings because I don’t know how to respond to them well. (ON2004) -I felt so angry at the time, but I didn’t show it to anyone. (ZL2819) -I was a little bit anxious there and afraid I was going to ruin everything, but I stayed like I am without showing emotion. (AA0716) -I don't want to express how I feel. (LZ2807) -There’s no point in expressing emotions, and this would only show the other person that we are “weak.” (P23011)	-One student mentioned that she doesn’t bother regulating her emotions and just waits for them to disappear on their own. (YF2) -I believe that male interaction in our session on emotional expression was weak, and I wished there was more in this regard (but given our society and stigma towards emotional expression among males, this was expected). (YF1) -The students’ comments in the training sessions make clear the need to fight stigma against emotional expression. (YL2) -Most of the participants stated that there was no point in expressing, let alone regulating, emotions, as this would only show the other person that we are “weak”. (YF1) -Also, many believed that there’s no need to express emotions because “no one will ever understand me.” (YF3) -Expressing sadness is viewed as weakness, particularly in men. (YF4)
(3) Emotional eating	- I ate and ate and ate. (AG4317) - I felt sad and disappointed because I know I can do better; after that I went and ate a croissant (I’m an emotional eater). (CR0310)	
**III. Unregulated Aggression (toward self and others)**		
(1) Physical abuse	-I did something that I haven’t done in a long time. I started hurting and cutting myself again. (CC1703) -I’m punching walls when I’m angry. (GN1507)	-Hitting and biting others and self (to feel pain) came up in the class discussion. (YF2) -Hurting others or breaking things to express anger or frustration are some examples students shared as to how they handle conflict. (YL1)
(2) Verbal Abuse	- I shout at everything and everyone. (AG1012) -When we fight, I yell at and punch everything. (GN1507) -I ended up shouting, screaming, blaming him, after all. (MT1707)	-Another discussion was around the example where one boy replies with hurtful words after the other says hurtful things to him; a few participants said this was an adaptive response since they shouldn’t let anyone “eat their head” [overpower them]. They believe that such a response ensures that “the other person will not think [they] are weak.” (YF2)

**Table A4.** Theme four—emotional regulation and coping/adaptive practices.

Subthemes:	Quotes from students:	Quotes from YLs and YFs:
**I. Functional Confrontation**		
(1) Engaging (with) the stress trigger/taking action to better the distressing situation	-While I was scared [for my health], I was always in my room, worried, thinking too much about it. In the end, I went to see the doctor… (SR1603) -I took part in sessions on leadership and other soft skills. I loved these. I feel so proud of myself. I’m working on being the better me. It feels good to look at the mirror now, and to not cry. (CS0104) -I started to focus on my work to study on time, so last week my grades rose significantly. (RR1909) -I’m working on myself to get good grades to get my phone back. (CC1703) -I went and talked to [my friend] and told him I’m upset with what happened; we came to an agreement and went back to how we used to be and the sadness went away. (GM0112)	
**II. Reorienting attention**		
(1) Cognitive reframing	-But then, I sat down and thought for a little bit: why am I complicating things? It’s just two days. I shouldn’t be worried. After that reflection, I got up, put a smile on my face and said to myself: let’s think positively. I spent the weekend enjoying my time with my family and my dog and making the most of it! (ML1812) -Later that night I practiced piano and I knew that I could do far more things than just get good grades. (CR0310) -[…]. Today I started seeing things in a different perspective and my body was less stressed. I think I started to feel like this because I changed the way I see things. (MC0605) -[…]. And after that I obliged myself to think positively. (CJ0164) -I gave myself a pep talk reassuring myself that everything would be okay. (AA0716) -Not giving up matters. I told myself that if you don’t give up and work hard, you always achieve. I didn’t let myself go down, so it feels I am happy. After I started to work really hard, I am continuing, and I will never stop. (RJ0612)	
(2) Distraction	-I would distract and distance myself from this feeling either through my phone or by talking to friends. (CG0207) -I went and watched TV and read my favorite book to make myself feel better. (CR0310) -I went to my room and drew to forget. (JS1005) -At the end of the stressful day, I decided to go to the *kermesse* [fair] to change my thoughts and mood. (CS0104) -Going to the étude [group study] gives me good vibes and I have fun away from the troubles of the day. (LB0803) -I feel better when I listen to music. (TP1311) -Whenever I start to get overwhelmed by these emotions, I listen to music, my happy place. (MC0605) -I draw because of the calmness that it fills me with. (GM0112) -I listened to some music to feel better. (PS8649)	
**III. Relaxing and/or Self-Soothing Techniques**		
(1) Crying	-I only cried and slept; I had cried during our calls and while chatting. (AG1012) -I cried a lot till […]. I froze for a while, not making any move, and felt calmer afterwards. (MT1707)	-After the mindfulness exercise, two girls were crying. It had proved cathartic for them. (YL1)
(2) Prayer	-I controlled [this] fear with prayer. (SR1603) -I prayed a lot to the Virgin Mary; what happened after was that I was shown a great sign and I stuck to my call even further and got out of the confusion. (SM271)	
(3) Physical activity and self-care	-My biggest relief and happiness is when I go to school to play basketball, my favorite activity, and this is the place I let go of my worries and tiredness and store positive energy in my body to go on with my life. (TP1311) -To change mood, I did something I love that relaxes me, such as fixing my hair and dressing up, then I continued my day in a fresher frame of mind. (ES1008)	
(4) Breathing	-Once I realized the state of anxiety I was in, I took several deep breaths, and I gave myself a pep talk—not sure if this is related to the breathing used in mindfulness, but it helped me deal with the anxiety. (AA0716)	
**IV. Other Emotional Management Resources**		
(1) Emotional Support	-When I’m sad my friends cheer me up and listen to me till the end, and I know they’re not fake. (LB0803) -Family problems happen pretty frequently. I mostly deal with these, but I am also able to share and discuss them with close people. (RN0907) -Maybe for some people our age, emotional support is an overlooked blessing—not for me. (CS0104)	
(2) Journaling	-I can’t express my emotions in any way other than writing what I feel (or crying). (CS2412)	-The benefit of the take-home diary exercise was to let things out and express what the learners wanted to say freely. (YF3) -Students said it helped them discover themselves and learn about personal issues they had to work on. (YF2)
